# Synergistic Effect of Thermosonication on the Stability of Bioactive Compounds and Antioxidant Activity of Blackberry Juice

**DOI:** 10.3390/foods14050901

**Published:** 2025-03-06

**Authors:** Cristiane Nunes da Silva, Juliana Rodrigues do Carmo, Bruna Vieira Nunes, Fernanda Demoliner, Vanessa Rios de Souza, Sabrina Carvalho Bastos

**Affiliations:** 1 Department of Nutrition, Federal University of Lavras, DNU/UFLA, Lavras 37203-202, MG, Brazil; kristtiane2015@gmail.com (C.N.d.S.); demoliner.fernanda@gmail.com (F.D.); 2 Department of Food Science, Federal University of Lavras, DCA/UFLA, Lavras 37203-202, MG, Brazil; juliana_docarmo@yahoo.com.br (J.R.d.C.); brunavieiranunes@gmail.com (B.V.N.); vanessardsouza@gmail.com (V.R.d.S.)

**Keywords:** fruit juice, anthocyanins, phenolic compounds, ultrasound, heat treatment

## Abstract

Thermosonication is a technique that combines ultrasound with mild temperatures and can be applied as an alternative to thermal pasteurization. This study aimed to evaluate the synergistic effect of thermosonication (Termo) on bioactive compounds (total anthocyanins concentration and total phenolic compounds), antioxidant activity and physicochemical characteristics of blackberry juice in comparison with conventional heat treatment (TT). The experiment was conducted based on the Central Composite Rotational Design, varying the amplitude (60% and 90%), temperature (64 °C and 86 °C) and time (114 s and 517 s) factors. The results showed that the amplitude and temperature factors significantly influenced (*p* < 0.05) the content of bioactive compounds studied, as well as the antioxidant activity and physicochemical properties, showing that the thermosonication treatment using 60% ultrasonic amplitude and 86 °C temperature provided more excellent retention and less degradation in the content of anthocyanins, phenolic compounds, antioxidant activity, and physicochemical properties (pH, acidity, total soluble solids and colour) of blackberry juice. Higher ultrasonic amplitude (90%) promoted changes in the physicochemical properties and degradation of the bioactive compounds studied and antioxidant activity. However, the limitations of this study are related to the specific matrix used, the seasonality of these fruits, the availability of raw material for processing and the limitation of large-scale ultrasonic equipment. These factors limit the expansion of these findings to other products. Overall, thermosonication can be considered a promising technique. Still, for its implementation as a possible alternative to conventional thermal methods, further studies are needed to investigate the stability of bioactive compounds and antioxidant activity of blackberry juice better.

## 1. Introduction

The consumption of fruit juice has grown significantly in recent years, mainly due to its pleasant sensory characteristics and because it is a good source of vitamins, minerals and several bioactive constituents, thus being a healthier alternative [1]. Blackberries are highly recognized for their richness in bioactive compounds. They stand out as one of the largest sources of phenolic compounds, particularly anthocyanins [2,3], the most abundant natural pigments in red fruits, especially blackberries [4,5]. Anthocyanins are responsible for the colour of these fruits and their high antioxidant potential, which can offer numerous positive impacts on health, such as in the prevention of cardiovascular, cancerous and neurodegenerative diseases [3,6]. Among the products obtained from these fruits, juice, jelly, and puree are the most appreciated due to their pleasant flavour, desirable sensory characteristics and high nutritional value [4,7,8].

Conventional heat treatment is one of the most widely used preservation methods in fruit-based beverages, which aims at food safety and shelf-life extension due to enzymatic inactivation and elimination of microbial load [9,10]. However, several studies have reported that heat is associated with the degradation of nutrients present in foods and changes in sensory characteristics and, consequently, loss in the quality of these products [11,12,13,14]. Phenolic compounds, especially anthocyanins, are among the bioactive compounds that suffer the most significant losses during conventional heat treatment, more incredible with the treatment’s rigour [11,15].

To obtain safe foods with appropriate shelf-life extension and meet consumers’ desire for healthier products, preservation techniques have been modified or developed to minimize the rigour of conventional methods, aiming to provide products of higher quality [16]. Ultrasound is a non-thermal method that promotes microbiological and enzymatic inactivation through cavitation, which involves the formation, growth and implosion of bubbles when a medium is subjected to irregular oscillations [17,18]. The collapse of these cavitation bubbles leads to mechanical and chemical effects related to local actions (pressures of up to 1.000 atm and temperatures of up to 5.000 °C), which promotes the disintegration of the cellular structures of microorganisms and enzymatic denaturation [19].

Despite the potential of this technology to replace traditional heat treatment by minimizing sensory and nutritional changes, ultrasound must be combined with other techniques, such as heat, to achieve satisfactory results since the use of ultrasound alone has not shown the same effectiveness compared to pasteurization in microbiological and enzymatic inactivation [16,20]. Several studies have shown that thermosonication can potentially increase the enzymatic and microbial inactivation rate, which is reflected in a reduction in the rigor of conventional heat treatment—time and temperature—and consequently minimizes nutritional changes and deterioration in food quality.

Thermosonication provided significant reductions in the activity of the polyphenol oxidase enzyme in peach juice [21] and strawberry juice [7], as well as in the inactivation of peroxidase in blueberry juice [22], spinach juice [23], and in the inactivation of pectin methyl esterase in guava juice [24]. In the spores of *Alicyclobacillus acidoterrestris* in apple juice [25], in *Escherichia coli* in spinach juice [23], in *Staphylococcus aureus* in orange juice [26] and in *Escherichia coli* and *Saccharomyces cerevisiae* in khoonphal juice [27] compared to conventional heat treatment. In addition to providing more excellent retention in the content of phenolic compounds, flavonoids, anthocyanins, ascorbic acid, antioxidant activity and colour of several types of juices, such as blueberry juice [22], verjuice [28], plum [29], current [30] and onion juice [31]. These studies show that thermosonication is a possible alternative to thermal pasteurization, a relevant and effective technique for future applications in the food industry.

Although several studies have demonstrated that the synergistic effect of ultrasound with heat (thermosonication) is an effective preservation technique for microbiological and enzymatic inactivation in fruit juices and nectars, studies investigating the impact of thermosonication on the nutritional and bioactive stability of foods are still limited [24,26,28,30]. Thus, the present study aims to explore raw material that has been studied little about the topics addressed but which presents a differential for being a matrix rich in bioactive compounds, mainly phenolic compounds and anthocyanins, considered potent natural antioxidants, which exert several beneficial effects on health by inhibiting/neutralizing oxidative reactions caused by free radicals. In addition, no studies are still exploring thermosonication and its potential impact on the physicochemical properties and stability of bioactive compounds (phenolic compounds and anthocyanins) and the antioxidant activity of blackberry juice. Some studies have shown that thermosonication can increase and release bioactive compounds from the cell wall in shorter process times, allow extraction to occur at milder temperatures and with less energy expenditure, increase extract homogeneity and provide products with more excellent freshness and safety. In addition, it enables higher-quality extracts due to more excellent retention of bioactive compounds and, consequently, their biological properties (antioxidant activity) [28,30,32].

On the other hand, due to the adverse effects of conventional heat treatment, associated with losses due to thermal degradation of bioactive compounds, such as phenolic compounds and anthocyanins, it is essential to search for emerging non-thermal green technologies, such as ultrasound, to preserve bioactive compounds and maintain the nutritional and sensory properties of fruit juices. In this context, the study of this technique will allow us to know the potential effects (negative, neutral or positive) and implement it safely as an alternative to conventional heat treatments. Therefore, the present study aimed to evaluate the synergistic effect of ultrasound with temperature (thermosonication) on the stability of bioactive compounds (total phenolic compounds and anthocyanins), the antioxidant activity, and the physicochemical characteristics of blackberry juice.

## 2. Material and Methods

### 2.1. Raw Material

**Figure 1:**
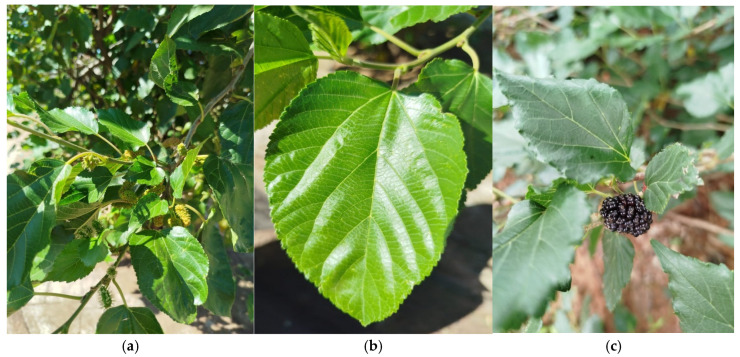
Blackberry plant and its green fruits (**a**); blackberry leaves (**b**), and fully ripe blackberry (**c**).

Fully ripe blackberries (*Rubus* spp.) were harvested manually in November 2022, during summer (21–28 °C) in the experimental orchard of the Fruit Sector of the Federal University of Lavras—UFLA (coordinates: 21°13′45.1″ S, 44°58′55.6″ W), Lavras, Minas Gerais, Brazil. The fruits were grown in soil classified as latosols (reddish or yellowish coloration, permeable and porous) according to the Brazilian Soil Classification System of Embrapa [33]. The fruits were immediately taken to the laboratory and selected based on the degree of ripeness and absence of physical damage, such as defects, malformations, or microbiological damage. Then, the blackberries were washed and sanitized for 20 min in a solution with 0.01% sodium hypochlorite. Afterwards, they were stored under freezing at −18 °C, protected from light, until the blackberry juice was prepared. Figure 1 illustrates the mulberry tree and its green fruits, the blackberry leaves and the blackberry at the stage of complete ripeness.

### 2.2. Preparation of Blackberry Juice

To obtain blackberry juice, the fruits were homogenized with water (ratio 3:7, *w*/*v*) in an industrial blender (Model 01). The juice obtained was filtered through a domestic sieve and then through the organza-type fabric to remove the solid parts. All batches of juice obtained were also homogenized to get a representative sample of all fruits. The juice was then stored in small plastic containers protected from light and froze at −18 °C until conventional heat treatment or thermosonication.

### 2.3. Experimental Designer

Based on the thermal treatments (binomials) usually used in fruit juices, reported in the literature [34,35,36,37], the temperature and time range of the study were initially defined from 60 °C to 90 °C and 30 s to 600 s. The experiment was realized according to the Central Composite Rotational Design (CCRD) 2^2^ + 5 central points + 2 axial points (−1.41 and +1.41) to evaluate the effect of the independent variables: temperature (X_1_) and time (X_2_) on the nutritional compounds and antioxidant activity of the blackberry juice. The 11 treatments defined by the design are described in Table 1.

### 2.4. Thermosonication

The thermosonication treatment as performed using a Q500 QSonica ultrasound machine (Ultronique, Indaiatuba, Brazil) equipped with a 1 cm diameter probe and a frequency of 20 kHz. The treatments were performed under two conditions (60% and 90% ultrasonic amplitude) under the time and temperature conditions described in the design shown in Table 1. Initially, the samples (50 mL of blackberry juice stored in a glass beaker) were preheated to the experimental temperature by immersing the samples in a water bath (Model Q215S2—Quimis, Diadema, Brazil) maintained at 95 °C.

Then, thermosonication occurred by immersing 0.5 cm of the probe in the glass beaker containing 50 mL of preheated blackberry juice. The temperature was monitored with a thermometer (Kasvi, Changsha, China) from the heating of the blackberry juice until the thermosonication treatment. A jacketed beaker with water circulation at the working temperature was used to maintain the temperature throughout the process. After each treatment, the samples were immediately cooled by immersing them in an ice bath and then frozen at −18 °C in glass tubes protected from light until the analysis.

### 2.5. Convencional Heat Treatment

Conventional heat treatment (TT) was performed by preheating the sample (50 mL of blackberry juice stored in a glass beaker) in a water bath (Model Q215S2—Quimus, Brazil) maintained at 95 °C. Upon reaching the temperature described in the design, the beaker containing the sample was transferred to a second water bath and adjusted to the working temperature when the treatment time was recorded. The temperature of the samples was monitored with the aid of a thermometer (Kasvi, Changsha, China) throughout the process (juice heating and heat treatment).

The thermosonication process resulted in a few degrees increase in temperature, mainly in the samples treated at the highest amplitude, requiring replication in the heat treatment. Therefore, the heat treatment was performed in two ways, the heat treatment 60 (TT 60) to reflect the thermosonication at 60% amplitude (Termo 60), and the heat treatment 90 (TT 90) the thermosonication at 90% amplitude (Termo 90). After the treatments, the samples were immediately cooled by immersion in an ice bath, stored in glass tubes protected from light and then frozen at −18 °C until analysis.

### 2.6. Physicochemical Characterization of Blackberry Juice

To characterize the fresh and treated blackberry juice (heat treatment and thermosonication), the pH, total soluble solids (TSS) and titratable acidity (TA) analyses were determined according to the methodology described by the Adolfo Lutz Institute [38]. The colour was determined using the parameters L* (lightness), C* (chroma value) and h* (hue angle) evaluated using a CIELAB colourimeter (CR-300 Chroma, Minolta, Japan) [39].

### 2.7. Bioactive Compounds

#### 2.7.1. Obtaining Extracts

The extracts for the analysis of anthocyanins, total phenolic compounds, and antioxidant activity (ABTS, DPPH, and β-carotene) were prepared according to the methodology described by Larrauri et al. [40]. 5 mL of blackberry juice was used and extracted sequentially with 40 mL of methanol with 50% water (50:50, *v*/*v*) at room temperature for 1 h with constant stirring. Afterwards, the supernatant was recovered, and 40 mL of acetone with 30% water (70:30, *v*/*v*) was added to the residue and extracted under the same previous conditions. The methanol and acetone extracts were combined, with the subsequent addition of distilled water, to obtain a volume of 100 mL.

#### 2.7.2. Determination of Anthocyanins

Total anthocyanins concentration were determined using the pH differential method [41]. The extracts were diluted in two buffer solutions with pH 1.0 and pH 4.5. The absorption was determined at 510 nm and 700 nm using a spectrophotometer. The total anthocyanin content in blackberry juice was calculated using the following equations.

*A* = (*A*_510nm_ − *A*_700nm_) pH_1.0_ − (*A*_510nm_ − *A*_700nm_) pH_4.5_ (1)

(2) $$TAC={\frac{A*MM*DF*10}{\epsilon *1}}^{3}$$

where *TAC* corresponds to the total anthocyanins concentration, *A* corresponds to the absorbance calculated by Equation (1), *MM* is the molecular mass of cyanidin-3-glucoside (449 g/mol^−1^), *DF* is the dilution factor, *€* the molar extraction coefficient (L mol^−1^ cm^−1^). The results were expressed in mg of cyanidin 3-glucoside equivalent per 100 mL (mg cyanidin 3-glucoside/100 mL).

#### 2.7.3. Determination of Total Phenolic Compounds

The total phenolic compounds (TPC) present in blackberry juice were determined according to the Waterhouse method [42]. Briefly, 0.1 mL of sample extract, 0.4 mL of distilled water, 2.5 mL of Folin-Ciocalteau solution (10%, *v*/*v*), and 2.0 mL of calcium carbonate solution (4%, *w*/*v*) were added. The samples were read using a spectrophotometer at a wavelength of 750 nm. The results were expressed in mg of gallic acid equivalent per 100 mL (mg GAE/100 mL of fresh weight). All readings for analyzing anthocyanins and total phenolic compounds were triplicate on the VIS 325–1000 nm spectrophotometer (Biospectro SP-22; Biospectro, Curitiba, PR, Brazil).

#### 2.7.4. Determination of Antioxidant Activity

The ABTS, DPPH and β-carotene methods subjected blackberry juices to antioxidant activity. The antioxidant activity by the ABTS^•+^ radical scavenging method was determined according to the methodology proposed by Re et al. [43], with samples reading in the spectrophotometer at 734 nm. The results were expressed in µmol of Trolox equivalent per 100 mL (µmol of Trolox equivalent/100 mL of fresh weight). The DPPH radical scavenging capacity was determined using the methodology of Rufino et al. [44]. The absorbance of the samples was read at 515 nm. The results were expressed in EC50, which refers to the amount of antioxidant necessary to reduce the initial concentration of the DPPH radical by 50% of the fresh weight of DPPH (EC_50_/100 mL of DPPH). The antioxidant activity by the β-carotene method was determined according to the methodology of Rufino et al. [45], with some modifications. Absorbance measurements were performed at 2 min and 120 min at 470 nm. The results were expressed as % inhibition of β-carotene oxidation. All readings of the antioxidant activity analyze (DPPH, ABTS and β-carotene) were performed in duplicate on a VIS 325–1000 nm spectrophotometer (Biospectro SP-22; Biospectro, Curitiba, PR, Brazil).

### 2.8. Statistical Analysis

All analyses were performed in triplicate, with respective means and standard deviations. The results were subjected to the multidimensional scaling technique to determine the similarity between thermosonication and heat treatment treatments. The transformation (Equation (3)) was carried out so that the responses were limited to a continuous scale of 0–1, making it possible to interpret them as indices, represented by y*_ij_*_,_ since the study variables had different scales.

(3) $$y_{ij}=\frac{y_{i}-minimum\left({y.}_{j}\right)}{maximum\left({y.}_{j}\right)-minimum\left({y.}_{j}\right)}$$

The similarity between treatments subjected to thermosonication and conventional heat treatment was determined by the multidimensional scaling technique, which obtains coordinate vectors represented by $y_{ij}=\left(y_{i1},\ldots ,y_{iq}\right)$, thus considering the Euclidean distance matrix $D=\left[d_{ij}\right]$ order *n* × *n* was formed from the data set for all variables. Each element of this matrix was calculated using the Euclidean distance defined by $d_{ij}^{2}={\left\|y_{i}-y_{i\prime }\right\|}^{2}$, para *i* ≠ *i*′, com *i* = *1*, *n*, where *n* is the number of treatments. Subsequently, correlated variables were excluded and a new dissimilarity matrix, defined as $\;∆=\left[\delta_{ii\prime }\right]$, is adjusted to consider fewer variables. Thus, the consideration of the proximity of each element, that is, ${d_{ii\prime }\approx \delta }_{ii\prime }$, was used as a validation criterion for the configuration of the stress function [46]. The non-metric multidimensional scaling defined by Jaworska Chupetlovska-Anastasova [47] and based on this algorithm considers the choice of a monotonic function to represent the relationship between the distance $d_{ij}$ e $\delta_{ij}$, which was applied. Kruskal described the algorithm used to minimize the stress function [48]. The biplot technique with predictive axes was used to validate the results to study the similarity between the treatment groups, considering a multivariate approach using the biplotGUI package from the R software (version 4.2.0) [49].

## 3. Results and Discussion

### 3.1. Physicochemical Characteristics of Blackberry Juice

**Figure 2:**
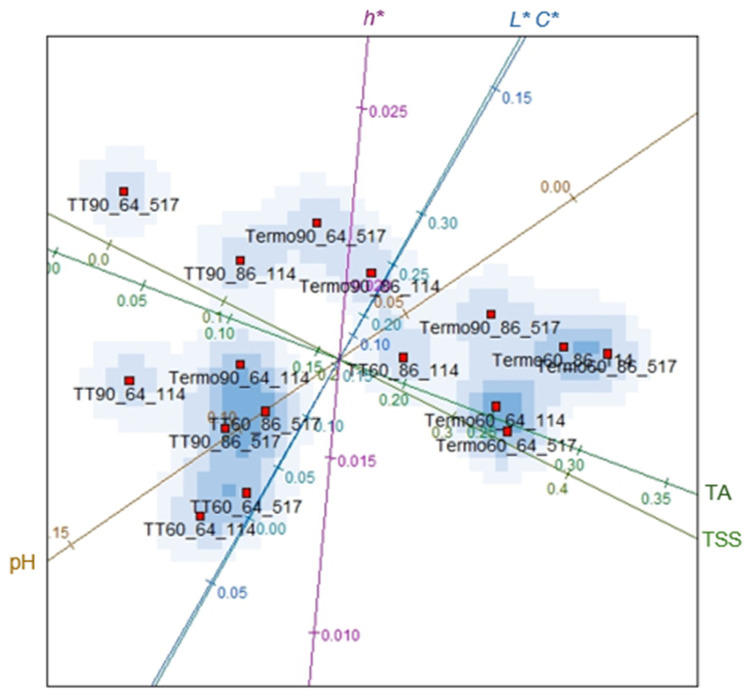
Biplots considering all variables: pH, total soluble solids (TSS), titratable acidity (TA), colour parameter: luminosity (L*), chroma value (C*), and hue angle (h*). 64 and 86 = temperature of 64 °C and 86 °C; 114 and 517 = time of 114 s and 517 s respectively. The lines with different colors in the figure indicate the evaluated parameters (pH, TSS, TA, L*, C* and h*).

In Figure 2, the variables related to the physical-chemical characteristics are described, namely pH, total soluble solids (TSS), titratable acidity (TA), colour parameter: luminosity (L*), chroma value (C*) and hue angle (h*). In this figure, treatments are grouped into regions delimited by the variables under study. Based on Figure 2, TT90_64_517 presented a more significant distinction from the other treatments, differing in the following variables: TSS and h*. The results showed a strong correlation between these parameters since the higher the h* values, the lower the concentrations of TSS. According to Tomadoni et al. [50], the reduction in TSS may be linked to the increase in h* values in the samples treated by heat treatment. In addition, higher values in the h* indicate a significant degradation in the colour of the blackberry juice subjected to TT90 (64_517). The treatments Termo90_64_114, TT60_86_517 and TT90_86_517 are indistinguishable regarding pH (0.10 and 0.20) and TA (0.10 and 0.15). The similarity in the scores highlights that the binomial at 86 °C for 517 s treated by heat treatment is accepted at 60% and 90% amplitude, demonstrating that these process conditions did not alter the pH and TA of the blackberry juice. These results, in turn, explain that heat treatment results in a greater significant reduction (*p* < 0.05) in pH. This fact indicates more considerable degradation of nutrients and better extraction of organic compounds by heat treatment since the increase in pH is strongly related to the degradation of nutrients that are sensitive to heat present in the juice [27,51]. A significant reduction (*p* < 0.05) in pH was reported in peach juice [21] and hazelnut milk [52] treated by heat treatment.

Termo90_86_517 is indistinguishable in terms of TA score (0.20 and 0.30) and pH (0.0 and 0.05). However, at Termo60, regardless of the time-temperature binomial, the samples presented groupings of treatments based on the TSS (0.3 and 0.4) and TA (0.25 and 0.30) indexes. These results showed that thermosonication increased blackberry juice’s pH, titratable acidity (TA), and total soluble solids (TSS). The results of this study were like those reported by Alves [53] and Souza [54] in grapefruit juice and camu-camu for TA. This increase may be due to the release of galacturonic acid from the pectin structure due to the cavitation phenomenon caused by ultrasonic waves [55]. Although the rise in temperature increases the degradation of bioactive compounds, applying ultrasound, combined with temperature, can enhance the sonolysis process of water molecules (OH^−^, H_2_O_2_ and H^+^), modifying the juice’s acidity [56]. The increase in TSS is due to cavitational waves, which cause damage to cellular structures, resulting in the formation of microscopic channels that improve the extraction of target compounds, thus enabling greater availability of TSS in the juice [57,58].

**Figure 3:**
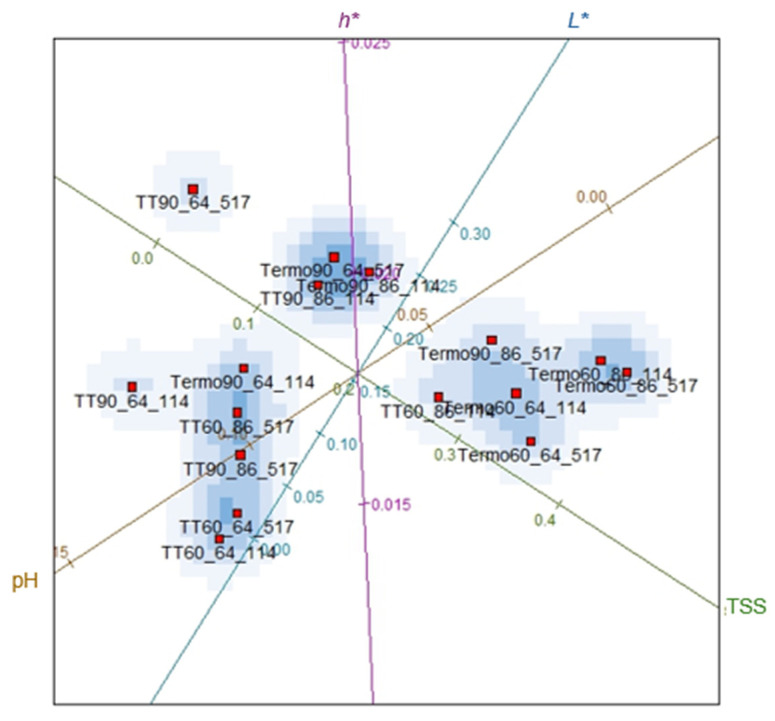
Biplots considering all variables: pH, total soluble solids (TSS), colour parameter: luminosity (L*), and hue angle (h*). 64 and 86 = temperature of 64 °C and 86 °C; 114 and 517 = time of 114 s and 517 s respectively. The lines with different colors in the figure indicate the evaluated parameters (pH, TSS, L* and h*).

**Figure 4:**
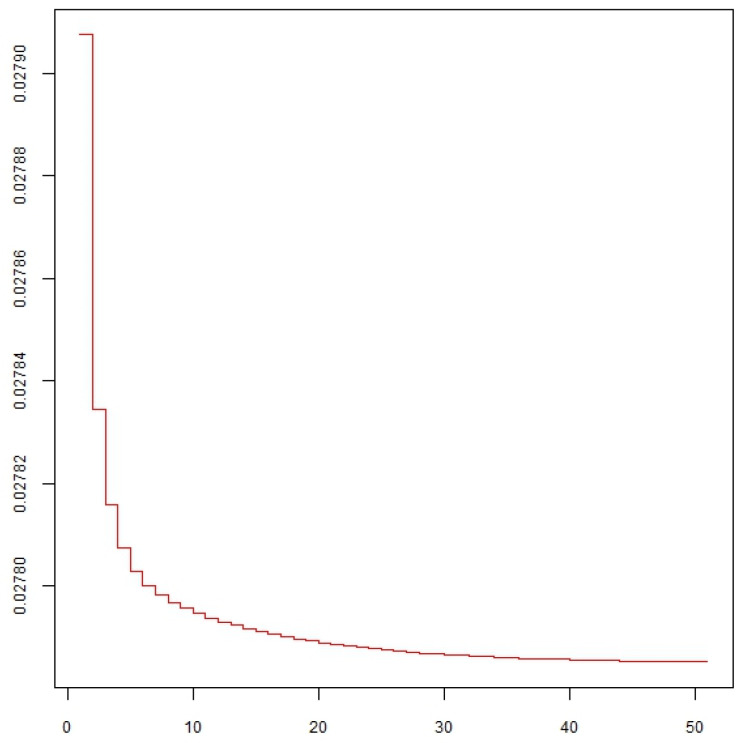
A graph of the stress function is calculated only with independent variables based on data on the physical-chemical characteristics of blackberry juice.

Considering all experimental variables, the degree to which all thermosonication and heat treatment treatments presented similarity was determined. Figure 2 shows that the variables TSS and TA, L* and C*, have a strong correlation, demonstrating that the homogeneity of these treatments should be evaluated in a way that considers only the independent variables. The results of this evaluation are illustrated in Figure 3. Regarding the validity of ordering the treatments into distinct sets, more information needs to be provided from an assessment of data based on all variables versus an evaluation that excludes correlated variables. The reduction of the variable is appropriate here since the graph of the stress function, shown in Figure 4, presented values close to zero. Thus, there is evidence that the statistical grouping determined by the independent variables is validated, without loss of information, for all variables illustrated in Figure 3.

To confirm the interpretations made previously, it can be observed in Figure 4, a tendency of grouping in the data points treated by thermosonication about TSS and pH, while in the thermal treatments, it was about TSS and L*. About h*, the treatments Termo90_64_517, Termo90_86_114 and TT90_86_114 led to an increase in the values of this variable. The results showed that the amplitude had a significant effect (*p* < 0.05) on h* with an increase at the highest amplitude level (90%). Furthermore, in the binomial of 86 °C for 114 s, both treatments, thermal or thermosonication, provided an increase in h* in the blackberry juice. Changes due to sonication are related to the cavitation phenomenon, which can induce color changes due to the acceleration of chemical reactions, increased diffusion rate, dispersion, formation of aggregated compounds (carotenoid isomerization and oxidation reactions) and particle breakage [59,60]. Higher h* values were reported in blackcurrant juice [61], guava [62] and orange [63] treated by sonication, and in strawberry juice [50] and spinach [23] subjected to heat treatment.

These results show a tendency for the variables to be grouped by the treatment method, such as heat treatment and thermosonication applied to the juice. The heat treatments showed more remarkable similarities in pH (0.10 and 0.00) and thermosonication in TSS (0.20 and 0.4). This demonstrates that thermosonication allows lower pH values and higher TSS. The changes in pH after sonication may be related to the generation of new organic compounds and phenolic compounds, resulting from the cavitation effect that stimulates the formation of chemical reactions and the release of these compounds into the aqueous medium [64]. The results of this study agree with those obtained by Baltacioglu [21] in peach juice and Sasikumar et al. [27] in khoonphal juice.

The higher results in TSS can be attributed to the extraction efficiency due to the disruption of cellular structures and more significant amounts of TSS in the juice [53,58]. Furthermore, thermosonication can cause changes in glucose and fructose content, thus resulting in higher TSS levels [65]. Increased TSS was also reported in previous studies in mixed carrot and grape juice [66], orange [67], and red grape juice [68], all thermosonicated. On the other hand, heat treatment presented higher pH values and lower concentrations of TSS. The same behaviour was reported by Mtaoua et al. [69], who observed an increase in pH (*p* < 0.05) and a reduction in TSS values in date juice. Similar pH results were found in Indian fruit juice after pasteurization [1]. During thermal processing, the Maillard reaction can occur, promoting the conversion of sugars into other compounds, thus reducing TSS [69,70].

### 3.2. Bioactive Compounds and Antioxidant Activity

**Figure 5:**
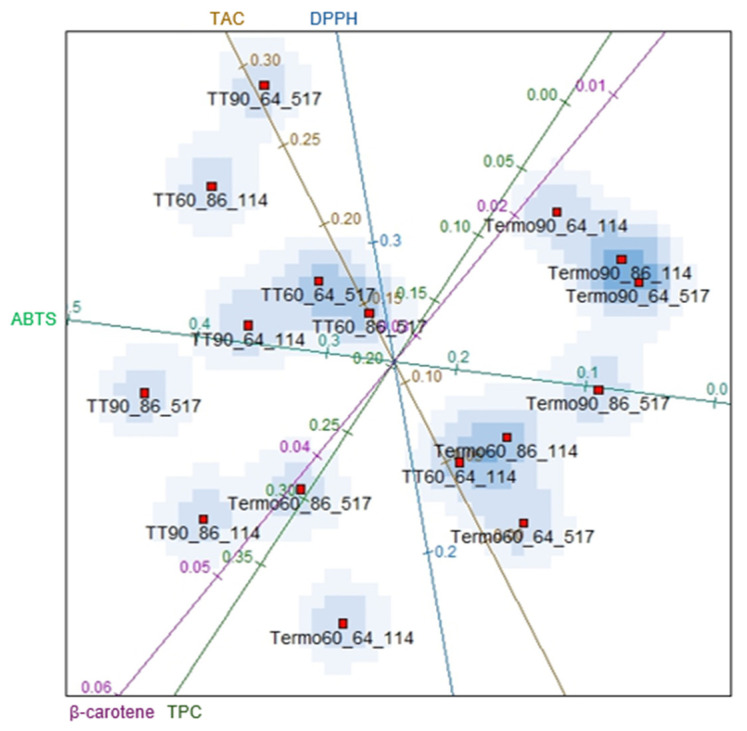
Biplots considering all variables: total anthocyanins concentration (TAC), total phenolic compounds (TPC), antioxidant activity ABTS, DPPH and β-carotene. 60 and 90 = amplitude of 60% and 90%; 64 and 86 = temperature of 64 °C and 86 °C; 114 and 517 = time of 114 s and 517 s, respectively. The lines with different colors in the figure indicate the evaluated parameters (TAC, TPC, ABTS, DPPH and β-carotene system).

The results were evaluated by multidimensional scaling of the interpretations of the generated biplots. Figure 5 describes the variables related to the bioactive compounds, namely: total anthocyanins concentration (TAC), total phenolic compounds (TPC), antioxidant activity by the ABTS method (ABTS), scavenging capacity of DPPH radical (DPPH) and by the β-carotene/linoleic acid system (β-carotene).

In Figure 5, the treatments are grouped into regions delimited by the variables under study (total anthocyanins concentration – TAC, total phenolic compounds – TPC and antioxidant activity by the methods ABTS, DPPH and β-carotene system). It is possible to observe the grouping of specific treatments, for example, for thermosonication in the following conditions: Termo90_64_114; Termo90_86_114 and Termo90_64_517 and in the thermal treatments: TT60_64_517 and TT60_86_517. These groupings demonstrate a similarity in the content of bioactive compounds (TPC and TAC) and antioxidant activity between the treatments. The similarities in the groupings treated by thermosonication were observed in the samples subjected to a higher level of ultrasonic amplitude (90%). The use of higher amplitude levels provides more significant intensification in the thermosonication process, thus generating more excellent ultrasonic effects on quality parameters, which may have contributed to the similarity in the number of bioactive compounds between treatments [71,72,73]. Regarding the thermal treatment, in addition to both treatments (TT60_64_517 and TT60_86_517) presenting the same amplitude level (60), which aimed to reflect thermosonication at 60%, the processing time (517 s) was also the same between the treatments studied. Time and amplitude can directly influence the content of bioactive compounds and antioxidant activity during product processing [73].

Thermosonication treatments showed greater significant differences (*p* < 0.05) about the following variables: antioxidant activity by the ABTS method (0.1 and 0.0), TPC (0.1 and 0.05), and antioxidant activity by the β-carotene method (0.02 and 0.01). The lowest coordinate values showed that thermosonication degraded the juice’s phenolics and antioxidant activity (ABTS and β-carotene). These losses can be attributed to sonochemical reactions generated through acoustic cavitation, with the formation of hydroxyl radicals that can enhance the hydrolysis of the glycosidic portions of phenolic compounds, thus resulting in the production of aglycones that have less stability compared to glycosides [74]. Furthermore, the formation of bubbles filled with water vapour and other dissolved gases, such as N_2_ and O_2_, can promote the oxidation degradation of TPC [72]. As for antioxidant activity, it may be associated with the reduction of TPC, resulting in a lower contribution to antioxidant activity [75]. These results corroborate with other studies, which observed decreased TPC and antioxidant activity in thermosonicated apple juice [75] and strawberry juice [7,76].

Analyzing the samples treated by heat treatment, the results showed that the most significant influences were on the variables ABTS (0.20 and 0.40), TAC (0.10 and 0.20), and DPPH (0.25 and 0.30). The results demonstrate that thermal treatment allowed higher levels of TAC, especially in samples treated at 64 °C/517 s (90) and 86 °C/114 s (60) compared to thermosonication. These results are different from those reported in previous studies, which observed a high thermal degradation of TAC, mainly at high temperatures (65 °C to 90 °C) in apple [75], tomato [77], and pomegranate [78] juice. Regarding antioxidant activity, the lowest amplitude level (TT60) resulted in the most significant capacity to eliminate DPPH radicals, representing more excellent antioxidant activity in blackberry juice. Ramírez-Melo et al. [79] observed more excellent antioxidant activity in beet juice treated with heat than in thermosonication. On the other hand, more significant values in antioxidant activity were found using the highest amplitude level (TT90) for the ABTS method. The structural rearrangement of TPC during heat treatment increases/reduction in antioxidant activity [80].

Termo90_86_114 and Termo90_64_517, and the treatments by Termo60_86_114 and TT60_64_114 showed similar levels of TPC, TAC, and antioxidant activity (ABTS method), respectively. Therefore, using any treatment among these groups can provide the exact TAC, TPC, and antioxidant activity in blackberry juice. The degree to which all treatments were similar was determined by considering all experimental variables (Figure 5). Based on Figure 5 it is possible to observe that the variables TPC (total phenolic compounds) and β-carotene (antioxidant activity) strongly correlate. This suggested that the homogeneity of these treatments should also be assessed in a way that only considers the independent variables.

**Figure 6:**
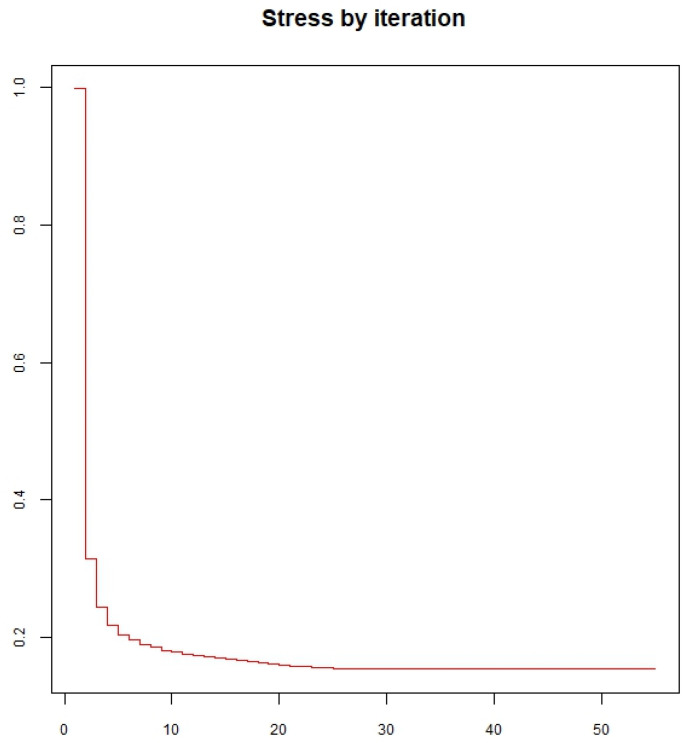
Graph of the stress function, calculated only with independent variables, from data on bioactive characteristics of blackberry juice.

**Figure 7:**
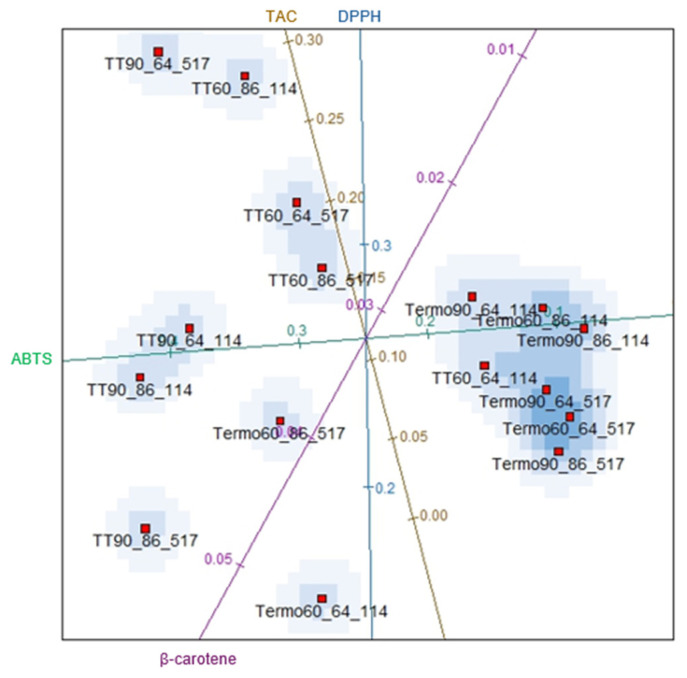
Biplots considering all variables: TAC (total anthocyanins concentration), antioxidant activity ABTS, DPPH, and β-carotene. 60 and 90 = amplitude of 60 and 90%; 64 and 86 = temperature of 64 and 86 °C; 114 and 517 = time of 114 and 517 s respectively. The lines with different colors in the figure indicate the evaluated parameters (TAC, ABTS, DPPH and β-carotene system).

Regarding the validity of ordering treatments into distinct sets, more information needs to be provided when moving from an evaluation of data based on all attributes vs. an assessment that excludes correlated attributes. Variable reduction is appropriate here, as the stress function graph (Figure 6) presented values close to zero. Thus, there is evidence that the statistical grouping determined by the independent variables is validated, without loss of information, for all variables illustrated in Figure 7.

To confirm the interpretations made previously, a tendency for clustering in the data points for the thermosonication and heat treatments can be observed in Figure 7. The most significant influence of thermosonication on the antioxidant activity (ABTS method) was in the Termo60_86_114 and Termo90_86_114 treatments, which presented lower concentrations, demonstrating that the combination of ultrasound with heat promoted the degradation of the antioxidant compounds in these conditions of time, temperature and amplitude studied, thus resulting in lower concentrations in the antioxidant activity in the blackberry juice. In addition, these results also show that the amplitude (60% and 90%) had no significant effect (*p* < 0.05) on the antioxidant activity and that the binomial at 86 °C and 114 s promotes more substantial degradation in the antioxidant compounds in both amplitudes studied (60% and 90%).

The treatments Termo60_64_114 (DPPH method) and Termo60_86_517 (β-carotene) were the conditions that presented the lowest losses in antioxidant activity after thermosonication treatment. It can be observed that the most significant retention of antioxidant activity for both methods (DPPH and β-carotene) was obtained at a lower level of ultrasonic amplitude. For anthocyanins, the highest content was observed in TT60_86_517, demonstrating that thermal treatment retains more anthocyanin pigments under these time and temperature conditions than thermosonication. It is worth mentioning that the highest TAC found in juices treated by thermosonication in some previous studies were at temperatures lower than those of the present study, 40 °C [27], 45 °C [81], 49.5 °C [82], 55 °C [83] and 60 °C [77] for the different types of juices. These results show that temperature has a significant effect (*p* < 0.05) on the TAC.

In general, the lowest degradation of bioactive compounds (TPC and TAC) and antioxidant activity was obtained when the samples were treated at a temperature of 86 °C and under an ultrasonic amplitude of 60% for thermosonication and heat treatment 60, which shows that the use of a lower ultrasonic amplitude provides more excellent retention of the bioactive compounds studied and, consequently, more excellent antioxidant activity. On the other hand, the use of a higher amplitude may hurt the bioactive compounds due to the provision of greater ultrasonic intensity (higher temperatures and pressures in the cavitation zone), thus resulting in the degradation of the bioactive compounds of blackberry juice and lower antioxidant activity [84]. The results of this study agree with those obtained for blueberry juice [85], bayberry [86] and hazelnut milk [52], which observed more excellent retention of bioactive compounds using lower ultrasonic amplitudes. However, the variability of the results obtained in the present study may be related to the specific matrix used (cultivation conditions, genetic variability, development stage), transportation and storage.

Blackberries are berries rich in bioactive compounds that can offer several benefits to human health due to their biological properties (antioxidant, anticancer, anti-inflammatory, anti-ageing and cardioprotective activity) [3,6]. The use of blackberries in the preparation of juice and other food products is an emerging field which aims to attract health-conscious consumers who are looking for healthy, high-quality, nutritionally rich and similar to natural products in the diversification of types and flavours of juices offered by the food industry; and in the development of the production of these fruits in producing regions, which allows not only a more significant economic return to the country, but also adds value to the undervalued product. Despite this, there is still a need for studies investigating the use of this technique on a larger scale and whether there is sufficient production for processing blackberries, aiming to evaluate the economic and technical viability and shelf life of thermosonicated products. On the other hand, for the application of thermosonication in other products, studies will be necessary to evaluate the composition and thermostability of the components of the different matrices.

## 4. Conclusions

In this study, blackberry juice was subjected to thermosonication to evaluate this technique’s effects on the quality parameters of the heat treatment. The results showed that the variables (temperature and amplitude) have a significant impact (*p* < 0.05) on the retention of bioactive compounds (total phenolic compounds and anthocyanins) and the antioxidant activity. In general, considering the CCRD treatments, it was observed that a temperature of 86 °C combined with a lower ultrasonic amplitude (60%) provides more excellent retention of phenolic compounds, anthocyanins and antioxidant activity, with the same behaviour observed for TT60. Higher amplitudes (90%) cause greater degradation of the studied compounds and more significant changes in blackberry juice’s physicochemical properties (increased pH and lower total soluble solids content). Further studies are needed to establish better conservation conditions for applying thermosonication as a possible alternative to traditional conservation methods. However, despite still being little explored, blackberry juice is a promising strategy, as it is an excellent source of phenolic compounds and anthocyanins with potent antioxidant activity. It is worth noting that the presence of bioactive compounds in fruit and vegetable juice improves the consumption of these products due to consumer concern for health and the search for a better quality of life. Therefore, studies with this matrix allow for adding value to the final product and developing regions that produce this fruit, providing the consumer with a healthy product with more excellent nutritional value and offering a new commercial product.

## Figures and Tables

**Table 1:** Coded and real variables for temperature and time factors according to the CCRD design.

Treatments	Temperature (X_1_)		Time (X_2_)	
	Coded	Real	Coded	Real
1	−1	64 °C	−1	114 s
2	−1	64 °C	1	517 s
3	1	86 °C	−1	114 s
4	1	86 °C	1	517 s
5	−1.41	60 °C	0	315 s
6	1.41	90 °C	0	315 s
7	0	75 °C	−1.41	30 s
8	0	75 °C	1.41	600 s
9	0	75 °C	0	315 s
10	0	75 °C	0	315 s
11	0	75 °C	0	315 s

## Data Availability

The data presented in this study are available on request from the corresponding author.
